# Mediterranean forested wetlands are yeast hotspots for bioremediation: a case study using azo dyes

**DOI:** 10.1038/s41598-018-34325-7

**Published:** 2018-10-29

**Authors:** Ana C. Sampaio, Rui M. F. Bezerra, Albino A. Dias

**Affiliations:** Departamento de Biologia e Ambiente (DeBA), Centro de Investigação e de Tecnologias Agro-ambientais e Biológicas (CITAB), Universidade de Trás-os-Montes e Alto Douro (UTAD), Quinta dos Prados, 5001-801 Vila Real, Portugal

## Abstract

Forested wetlands are interfaces between terrestrial and aquatic environments. These ecosystems play an important role in the hydrology, chemistry and biodiversity maintenance. Despite their high microbial diversity, there has been a lack of attention to the potential of their yeast communities. The purpose of this study is to evaluate the potential of yeasts isolated from a Mediterranean forested wetlands in decolorizing azo dyes. Azo dyes are synthetic, and highly recalcitrant to degradation. Ninety-two out of 560 isolates were randomly chosen to assess their ability to decolorize five azo dyes. Hierarchical clustering based on medium color changes during incubations was used to evaluate the isolates’ decolorization performance. All of the isolates that best degraded the 5 dyes tested were identified as Basidiomycota (Filobasidiales, Tremellales and Sporidiobolales). This work identifies new azo dye-degrading yeast species, and supports the hypothesis that forested wetlands are a niche for yeasts with bioremediation potential - namely azo dyes removal.

## Introduction

Forested wetlands are interfaces between terrestrial and freshwater systems. These areas, naturally flooded or saturated by surface or groundwater, support a significant component of woody vegetation that is well adapted to flooded or poorly aerated soils^1^. Flooded forests are particularly important in global hydrology, in the carbon cycle, in nutrient filtration, as carbon sinks, and in maintaining biodiversity^2,3^. They are one of the most fragile and threatened ecosystems in the world. Between 1896 and 1996, 94% of the original wetlands in central-western Iberian Peninsula become fragmented or disappeared completely^4^. In forested wetlands energy flow, which influences organic matter decomposition, is regulated by the hydroperiod^1^, with periods of aerobic/wet alternating with anoxic/dry conditions. The length of those periods, together with low temperatures, leads to partial decomposing of organic matter which may accumulate over time.

Among the saprophytic community, fungi play an important role in degrading recalcitrant biopolymers^5^. Yeasts are abundant and frequently isolated from flooded forests such as peatlands, floodplains or humid forests^6–8^ and, with regards to plant litter decomposition, are currently considered as pioneers^6,9^. The most prevalent yeast species found in wetlands belong to *Candida*, *Debaryomyces* and *Pichia* (Ascomycota), *Cryptococcus*, *Rhodotorula*, *Sporobolomyces* and *Trichosporon* (Basidiomycota)^6^. These genera include species capable of dye biotransformation, such as *Candida zeylanoides*^10^, *Candida tropicalis*, *Debaryomyces polymorphus*^11^, *Saccharomyces cerevisiae*^12^, *Candida krusei*^13^, *Candida oleophila*^14^, *Candida boidinii*^15^, *Cyberlindnera saturnus* and *Barnettozyma californica*^16^, *Scheffersomyces spartinae*^17^ and *Pichia occidentalis*^18^ among the ascomycetous yeasts.

Some basidiomycetous species, such as *Bullera armenica*, *Cryptococcus victoriae*, *Rhodotorula glutinis*, *Rhodotorula laryngis*, *Bullera armeniaca*, *Pseudozyma rugulosa*, *Trichosporon multisporon*, *Trichosporon akiyoshidainum* and *Trichosporon porosum*^13,19,20^, are able to biodegrade and decolorize several azo dyes. These dyes are an important group of synthetic colorants, termed xenobiotic compounds, which are highly recalcitrant to biodegradation processes. Besides their intrinsic toxicity, they have a negative environmental impact due to the potential formation, under anaerobic conditions, of aromatic amines which are viewed as carcinogenic and/or mutagenic^21^.

Despite biodiversity having received much attention from investigators, studies devoted to microbial diversity, particularly yeasts, remain scarce. This has had an impact upon the extent of knowledge regarding the ecological role of these microorganisms, their physiology and biochemistry and, consequently, their potential for biotechnological applications. When compared to bacteria and filamentous fungi, yeasts exhibit some attractive characteristics. Although not as rapidly as bacteria, yeasts can grow faster than most filamentous fungi, and, like the latter, they have the capacity to resist unfavorable environments^13^.

Based on the fact that yeasts are frequently isolated from forested wetlands and that some of them (*Cryptococcus*, *Candida*, *Rhodotorula*, *Pichia*, *Trichosporon*) are abundant in wastewaters^19,22^, and capable of decolorizing azo dyes by biodegradation^11,14,20,23^ it was hypothesized that forested wetlands may be reservoirs of biotechnologically-important wild yeast isolates. To test this hypothesis, for future use in bioremediation, yeasts previously isolated from decomposed leaves in a natural forested wetland were tested for their ability to degrade several azo dyes and the most promising isolates with high color(s) removal potential were identified using conventional-PCR and nucleotide sequence analysis.

## Methods

### Origin of yeast isolates

Yeast isolates were obtained during a leaf litter decomposition study in a freshwater forested wetland at Alpiarça^8^, a site located in the river Tagus hydrographic basin (southwest of Portugal) with an average altitude of 17 m, and a mean annual temperature and precipitation of 15.9 °C and 715 mm, respectively.

The site is waterlogged from October to June, and the dominant vegetation cover is willow (*Salix atrocinere*a Brot.) and alder buckthorn (*Frangula alnus* Mill.). In addition to deciduous trees and shrubs, the site has peat deposits and a floor covered by mosses. The main physicochemical parameters of superficial water in January and May can be found in the Supplementary Table S1. Cultivable yeasts from decomposed leaves of alder and willow were isolated over 64 days^10^. Pure cultures of yeast isolates were obtained and stored at −70 °C, in yeast malt extract broth (YMB; 0.3% malt extract, 0.3% yeast extract, 1.0% D-glucose and 0.5% peptone) supplemented with 30% (v/v) glycerol. From a universe of 560 isolates selected based on different colonial morphologies, 92 yeast isolates were randomly chosen to assess their ability to decolorize azo dyes.

### Azo dyes

Five azo dyes were used (Supplementary Table S2): Reactive Black 5, RB5 (C.I. 20505), Reactive Orange 16, RO16 (C.I. 17757), Reactive Violet 5, RV5 (C.I. 18097), Acid Red 57, AR57 (C.I. 15850) and Methyl Orange, MO (C.I. 13025).

Reactive Black (RB5) has a diazo structure, while the other dyes have a mono-azo structure. In addition to the azo bond(s) (–N=N–) as chromophore, all dyes have at least one auxochrome, such as amine (–NH_2_), sulfonate (–SO_3_H) and hydroxyl (–OH). Additionally, Reactive Violet 5 (RV5) contains copper in its composition. The textile dyestuffs RB5 and RV5 were kindly provided by DyStar Anilinas Têxteis Lta (Portugal) and Acid Red 57 (AR57) by Clariant Lta (Portugal). The dyes Reactive Orange 16 (RO16) and Methyl Orange (MO) were purchased from Sigma-Aldrich.

### Decolorization screening

The screening of the yeast isolates capacity to decolorize dyes was performed in an MM broth with the following composition (per liter): 5.0 g D-glucose, 1.0 g (NH_4_)_2_SO_4_, 1.0 g KH_2_PO_4_, 0.5 g MgSO_4_.7H_2_O, 0.1 g yeast extract, 0.1 g CaCl_2_.2H_2_O, and the respective azo dye at a final concentration of 50 mg L^−1^. Overnight yeast suspensions (Abs_640_ = 1.0) grown in YMB were used to inoculate autoclaved (121 °C, 15 min) 50-mL Erlenmeyer flasks containing 30 mL of minimal broth medium (MM). Color changes from two replicates per isolate and per dye were checked every 12 hours during 5 days of incubation, and data was only considered when it was the same for both replicates, otherwise the assay was repeated. Incubations were carried out in the dark, using an orbital shaker set a 120 rpm. Non-inoculated controls were included in all experiments.

### Bioaccumulation screening

Bioaccumulation of dyes onto yeast isolates was assayed in Erlenmeyer flasks (50-mL capacity) during the decolorization screening. At the end of the experiment, yeast suspensions were allowed to settle and the color of yeast biomass was visually checked. In addition, samples (5 mL) were taken and centrifuged 10 min at 4000 g. Supernatants were passed through a 0.22 µm filter and dye presence inspected (visually and by spectrophotometry), while the sediments (yeast cells) were washed with distillated water and centrifuged as above, the process being repeated three times, before observation of the sediment by naked eye and under the microscope.

### Data analysis of decolorization screening

The ability of yeast isolates to decolorize each one of the five azo dyes was analyzed using the Unweighted Pair Group Method with Arithmetic Mean (UPGMA), based on the Bray-Curtis similarity percentage. The data matrix was constructed using qualitative color change values. For example, during the incubation of 92 yeast isolates in MM supplemented with the azo dye RB5 over a time-course of 72 h, color changes was assigned as follows: initial black color (0), deep blue (1), light blue (2), purple (3), light pink (4), white (5) and colorless (6), corresponding to zero (0), partial (1 to 5) or total decolorization (6). The resulting data matrix was analyzed using PRIMER V6^24^ (Clarke and Warwick, 2001).

### Identification of dye-decolorizing yeast isolates

For the identification of the yeast isolates with high decolorization ability (AGG 577, AGG 598, AGG 637, AGG 667; AGG 691, AGG 707, AGG 711, AGG 721, AGG 726; AGG 729 and AGG 730) total DNA from isolates was extracted^25^ after culture growth on MYP agar (malt extract 0.7%, yeast extract 0.0 5%, soytone 0.25%, and agar 1.5%).

DNA was amplified through conventional-PCR using the universal fungal rDNA ITS5 (5′-GGA AGT AAA AGT CGT AAC AAG G-3′)^26^ and LR6 (5′-CGC CAG TTC TGC TTA CC-3′)^27^ primers. Cycle sequencing of the D1/D2 domain (a fragment of 600–650 base pairs at the 5′-end of the 26S rDNA) employed both forward NL1 (5′-GCA TAT CAA TAA GCG GAG GAA AAG-3′) and reverse NL4 (5′- GGTCCG TGT TTC AAG ACG G-3′) primers^28^. Sequences were obtained with an Amersham Pharmacia ALF express II automated sequencer by Biopremier, Portugal.

The sequences obtained were used for a BLAST search for similar sequences in the NCBI/GenBank Data Library, and registered under the accession numbers JQ429326 for AGG729 isolate and JQ917424 – JQ917433 for the other isolates. For tree construction reference, sequences were retrieved from the GenBank database (on 16/05/2018), and aligned using the ClustarW algorithm^29^. A phylogenetic tree was computed using the Maximum Composite Likelihood method for genetic distances, and the Neighbor-Joining as a grouping method. Gaps were deleted and bootstrap values were calculated from 1,000 replications. Finally, the alignments and the phylogenetic tree were run in MEGA 6.06^30^.

## Results and Discussion

### Screening of the decolorizing ability of yeast isolates in liquid media

**Figure 1:**
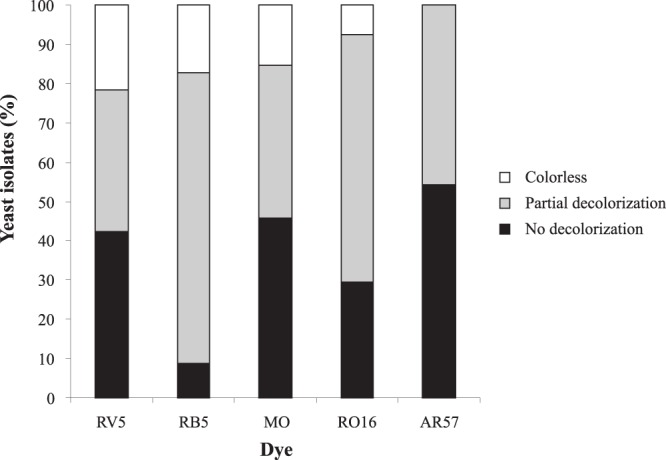
(In)ability of 92 yeast isolates to decolorize the dyes RV5, RB5, MO, RO16 and AR57, after 5 days of incubation in liquid medium.

Dye-decolorizing ability of yeast isolates was first evaluated using MM supplemented with each of the five azo dyes tested (RB5; RO16; RV5; AR57 and MO). Yeast isolates differed markedly in their ability to decolorize the tested dyes (Fig. 1). Forty three isolates (~47%) were able to decolorize at least one azo dye, which is a result superior to that observed in yeasts isolated from Las Yungas rainforest (~39%) in Argentina^16^. None of the yeast isolates was able to decolorize AR57 completely, while total colorless media were obtained by 7.6% (RO16), 15.2% (MO), 17.4% (RB5) and 21.7% (RV5) of the isolates.

The highest percentages of the isolates that totally failed to remove color was achieved in MM supplemented with AR57 (54.3%), followed by MO (45.7%) and RV5 (42.4%). For these yeast isolates the dyes AR57, MO and RO16 were the most recalcitrant. Paradoxically, RV5, also a monoazo and sulfonate dye with cooper in its structure, exhibited the highest percentage of total decolorization (~22%), followed by RB5 (~17%), a diazo and sulfonate dye. RB5 and RO16 had high percentages of partial decolorization. RB5 was the dye with more visible colored intermediates generated during the assays.

**Figure 2:**
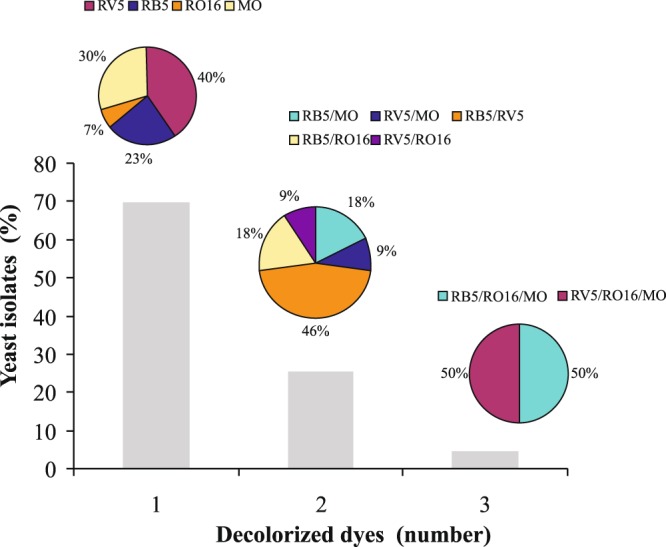
Percentage of the yeast isolates able to decolorize completely the media (n = 43), for one, two or three dyes. For each situation the percentage of dyes or their combination are also shown.

The majority (69.8%) of the yeast isolates were able to decolorize completely a single dye, 25.6% managed to decolorize two dyes, and only 4.7% three dyes (Fig. 2). This could indicate that bioprocesses involved in dye degradation are highly dependent on dye structure and the yeast species or strains involved. The chemical structure and design, the number of azo bonds, the type of other functional groups (amine, carboxyl, sulfonate and hydroxyl) and the presence of chloride and metals influence biodegradation^31–33^. In general, monoazo dyes decomposed rapidly, but the monoazo dyes tested in the present work appeared more recalcitrant to degradation by these yeast isolates than RB5, a diazo dye. Also, Reactive Blue 221 (a monoazo and cooper dye) was rapidly decolorized by yeast isolates, followed by RB5 (a diazo dye) was reported by Martorell *et al*.^16^. Compared to Reactive Yellow 84, RB5 (another diazo dye) was more easily decolorized, which is possibly due to its simpler structure, without triazine heterocycles or chloride, and its lower molecular mass^16^.

With regards to individual dyes, RV5 was the easiest to completely decolorize by yeasts, followed the descending order by RB5, MO and RO16. Amongst the yeasts to be able to decolorize two dyes, the most frequent combination was RV5/RB5 (46%), followed by RB5/MO and RB5/RO16 (both with 18%) and RV5/MO and RV5/RO16 (both with 9%). This pattern supports the assumption that yeasts exhibit, at least to some degree, substrate preferences despite the broad substrate specificity of mainly enzymes associated with azo-dyes decolorization, such as azoreductases, manganese-dependent peroxidases and tyrosinases^33^. Furthermore, other enzymes may be involved in this process. Recently, NADH-DCIP reductase was reported in *Pichia kudriavzavii* (formely *Issatchenkia orientalis*) during biodegradation of RO16^34^.

In the present work, only two isolates showed the ability to completely decolorize three dyes: AGG 707 (RV5/MO/RO16) and AGG 730 (RB5/MO/RO16). The latter isolate also exhibited the ability to decolorize AR57 almost completely.

**Figure 3:**
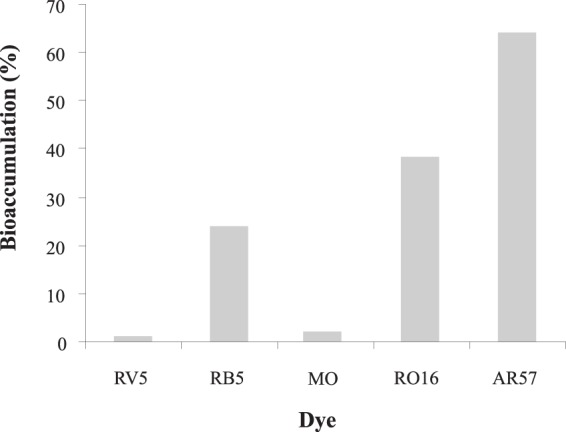
Percentage of yeast isolates that present biomass dye adsorption. The percentage relates to the isolates able to decolorize totally or partially, the dyes RV5 (n = 53), RB5 (n = 84), RO16 (n = 65), MO (n = 50) and AR57 (n = 42).

Ranging from 1.9% to 64.3%, bioaccumulation (Fig. 3) contributed to color removal by the yeasts isolates, depending on the dye used. For example, for AR57 dye, bioaccumulation was responsible for dye decolorization in the majority of the isolates. This mechanism was also important in the removal of RO16 and RB5, while for RV5 and MO, the cause was residual. Taking into account both the supernatant decolorization and the suspended cells, the decolorization of dyes RV5, MO and RB5 was mainly caused by biodegradation (transformation), in contrast to dyes AR57 and RO16, which were mainly removed through sorption.

**Figure 4:**
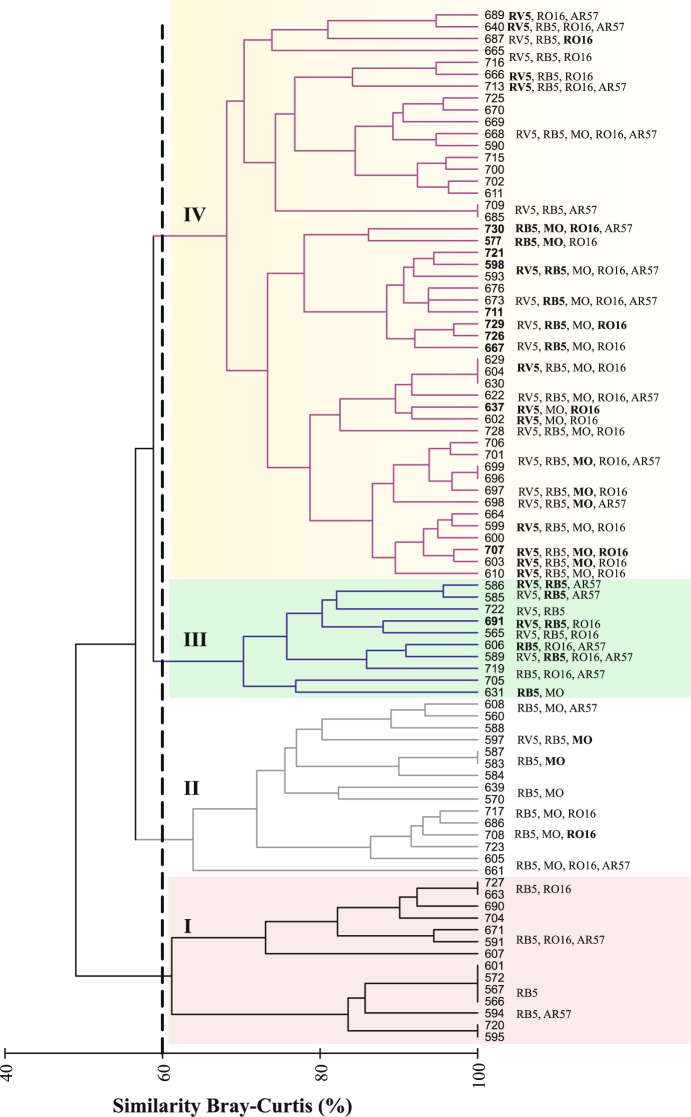
Dendrogram for hierarchical clustering (using UPGMA) of yeast isolates (n = 92) abilities in decolorizing 5 dyes, based on the Bray-Curtis similarity matrix. The four groups of yeast isolates separated at a 60% similarity threshold (dotted line) are indicated by Roman numeral: I isolates with low or zero decolorizing abilities; II isolates that partially decolorize some dyes and/or totally decolorize one dye (MO, RO16 and AR57); III isolates that partially or fully decolorize RV5 and/or RB5; IV isolates with a wide range of decolorizing abilities. Numbers in boldface correspond to the AGG isolates identified in this study.

The cluster analysis of all yeast isolates’ decolorizing abilities (Fig. 4), at a 60% similarity threshold, indicated four groups (I, II, III, IV) of yeast isolates: a) I grouped isolates with low or zero decolorizing abilities; b) II aggregated isolates capable of partial decolorization of some dyes and/or total decolorization of one dye (MO, RO16 and AR57); c) III clustered isolates with the ability to partially or fully decolorize RV5 and/or RB5; d) IV grouped the majority of the isolates (n = 48) with a wide range of decolorizing abilities, in terms of number of dyes and levels of decolorization.

These results indicate that the biodegradation of azo dyes depends not only on the chemical structure of the dye, but also on microbial diversity. Moreover, the distinct azo-dye decolorization capacities exhibited by the yeast isolates, were probably due to the physicochemical complexity of substrates, and the gradient of nutrients and oxygen found in the forested wetlands from which the yeasts originate^6–8^.

### Identification of the dye decolorizing yeast isolates

Based on two criteria: (i) the number of the dyes decolorized and (ii) on the mechanism of discoloration (degradation *versus* accumulation), the most promising yeast isolates were selected.

**Figure 5:**
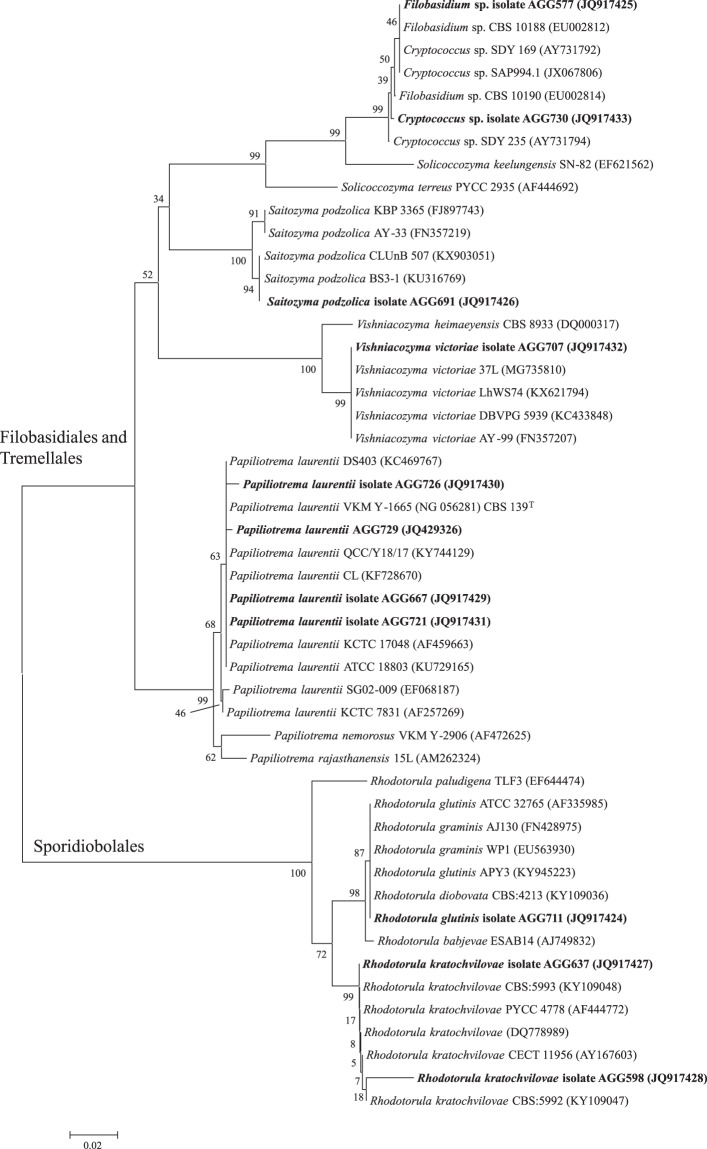
Phylogenetic tree of selected isolates obtained by Maximum Composite Likelihood method for genetic distances and the Neighbor-Joining as grouping method, of 26S rDNA gene (D1/D2 domains). Bootstrap percentages from 1,000 replicates are shown in each node. Scale bar indicates number of differences. Sequences obtained in the present study are in boldface. Additional sequences were retrieved from GenBank (species names followed by strains or isolates references and the corresponding accession number in brackets).

Following these criteria, from the initial 92 isolates, 11 were chosen, ten from group IV and one from group III (Fig. 4). All the selected isolates AGG 577, AGG 598, AGG 637, AGG 667, AGG 691, AGG 707, AGG 711, AGG 721, AGG 726, AGG 729 and AGG 730, belong to the Basidiomycota phylum^35,36^ (Table 1 and Fig. 5): (a) four isolates of *Papiliotrema laurentii* (Kuff.) X.Z. Liu, F.Y. Bai, M. Groenew. & Boekhout, comb. nov. MycoBank MB813295 (2015); (b) one isolate of *Vishniacozyma victoriae* (M.J. Montes, Belloch, Galiana, M.D. García, C. Andrés, S. Ferrer, Torr.-Rodr. & J. Guinea) X.Z. Liu, F.Y. Bai, M. Groenew. & Boekhout, comb. nov. MycoBank MB813285 (2015); (c) one of *Saitozyma podzolica* (Babeva & Reshetova) X.Z. Liu, F.Y. Bai, M. Groenew. & Boekhout, comb. nov. MycoBank MB813373 (2015); (d) two isolates of *Rhodotorula kratochvilovae* (Hamam., Sugiy. & Komag.) Q.M. Wang, F.Y. Bai, M. Groenew. & Boekhout, comb. nov. MycoBank MB813353 (2016); (e) one isolate of *Rhodotorula glutinis* (Fresen.) F.C. Harrison (1928); and (f) two isolates belong to *Cryptococcus* and/or *Filobasidium*. According to the phylogenetic tree based on D1/D2 domains of 26S rDNA gene (Fig. 5) the closest relatives of *Filobasidium* AGG 577 are *Cryptococcus* sp. SDY 169 (AY731792), *Cryptococcus* sp. SAP994.1 (JX067806), and *Filobasidium* sp. CBS 10188 (EU002812), with 99% identity (number of bases in BLAST match/number of bases in the sequence – 514/515). For *Cryptococcus* sp. AGG 730 the highest identity percentage obtained by BLAST 99%, was also for *Cryptococcus* sp. SAP994.1 (614/616), followed by *Cryptococcus* sp. SDY 235 (AY731794) (590/592) and *Filobasidium* sp. CBS 10188 (EU002812) (592/595). The isolate *Cryptococcus* sp. AGG 730 also diverges 2 base pairs from *Cryptococcus* sp. SDY 169 (AY731792). Yeast isolates most related to AGG 577 and AGG 730 were from distinct environments in the Mediterranean: phylloplane^37^, São Domingos mine^38^ and nectar from flowers in Andalusian woodlands^39^.

**Table 1:** Dye decolorization after 120 h of incubation in MM by yeast isolates. Zero decolorization (−); partial decolorization (+ and ++); complete decolorization or colorless (+++).

Isolate	RB5	RV5	RO16	AR57	MO	Accession number	Identification (%)
Dye							
AGG 577	+++	—	+	—	+++	JQ917425	*Filobasidium* sp.
AGG 598	+++	+++	++	—	++	JQ917428	*R. kratochvilovae* (99%)
AGG 637	—	+++	+++	—	++	JQ917427	*R. kratochvilovae* (98%)
AGG 667	+++	+	++	—	+	JQ917429	P. laurentii^*^ (100%)
AGG 691	+++	+++	++	—	—	JQ917426	S. podzolica^**^ (100%)
AGG 707	++	+++	+++	—	+++	JQ917432	V. victoriae^***^ (100%)
AGG 711	+++	++	+	+	++	JQ917424	*R. glutinis* (100%)
AGG 721	+++	+++	++	+	++	JQ917431	*P. laurentii* (100%)
AGG 726	+++	+	+++	+	+	JQ917430	*P. laurentii* (100%)
AGG 729	+++	+	+++	+	++	JQ429326	*P. laurentii* (99%)
AGG 730	+++	—	+++	++	+++	JQ917433	*Cryptococcus* sp.

Yeast isolates identification based on D1/D2 domain of the 26S rDNA region BLAST in NCBI/GenBank and respective accession numbers. ^*^Formerly *Cryptococcus laurentii*; ^**^Formerly *Cryptococcus podzolicus*; ^***^Formerly *Cryptococcus victoriae*.

Interestingly, the yeast isolates AGG 667, AGG 726 and AGG 729, grouped by the cluster analysis based on dye decolorizing abilities (Fig. 4) belong to the same species: *P. laurentii* (Fig. 5). Also, the isolates AGG 577 and AGG 730, clustered by UPGMA based on azo dyes degradation profiles, are taxonomically close (Filobasidiales order). In parallel, isolates AGG 707 and AGG 691 from different species, had distinct dye decolorization profiles and azo-dye decolorizing profiles positioned them in different UPGMA clusters. Nevertheless, isolates AGG 598 and AGG 637, although belonging to the same species (*R. kratochvilovae*) had different patterns in decolorizing dyes. These two isolates differ from each other in 5 base pairs (505/510) and are probably two different strains. As pointed out previously, dye degradation capabilities are not only species-dependent, but also strain-dependent. In twelve isolates of *V. victoriae* (formerly *Cryptococcus victoriae*) only two were capable of dye decolorization^20^.

All the selected isolates were identified as basidiomycetous, which is probably a consequence of the environment from where the yeasts were isolated, decomposing leaves being rich in lignocellulosic compounds^6,40^. Enzymes involved in the breakdown of plant polymers or monomeric products from lignin degradation, have been associated with azo dyes degradation^11,15,16,19,23^ and some of them have been previously reported as synthetized by the yeast species identified in the present work^20,41^.

The majority of published works concerning azo dye color removal have involved mainly ascomycetous species^42^, despite some reports of using basidiomycetous yeasts^13,16,19,20,23^. Most of the basidiomycetous yeast species reported in dye decolorization are scattered in the orders Ustilaginales and Trichosporonales. More recently Rovati *et al*.^20^ have reported the ability to decolorize azo dyes in two Tremellales (*B. armenica* and *V. victoriae*) and in two Sporidiobolales (*R. glutinis* and *R. laryngis*) species isolated from soils from King George Island (Antarctica).

The present research, as in Rovati *et al*.^20^, identifies *R. glutinis* (Sporidiobolales) and *V. victoriae* (Tremellales) as amongst the isolates that had a high overall performance in azo-dye degradation. In addition, two new species of Tremellales (*P. laurentii* and *S. podzolica*), one species of Sporidiobolales (*R. kratochvilovae*) and two isolates from Filobasidiales are recognized as species with bioremediation potential for azo-dyes degradation. Other dark-colored effluents such as winery and pulp mill wastewaters were partially degraded by *S. podzolica* AGG 691^43^ and *P. laurentii* AGG 726^44^.

## Conclusions

In conclusion, yeast isolates differed markedly in their ability to decolorize the tested five dyes, and the majority completely decolorized a single dye. When two dyes were decolorized, the most frequent combination was RV5/RB5. Only two isolates had the ability to decolorize three dyes: AGG 707 (RV5/MO/RO16) and AGG 730 (RB5/MO/RO16). Bioaccumulation was the preferential mechanism of color removal used for AR57 and RO161. The isolates with high capacity for azo dyes remediation were distributed across three Basidiomycota orders: Tremellales, Sporidiobolales and Filobasidiales.

The ability of yeasts to remove azo dyes is dependent on dye structure, species and, within species, may vary among strains. This work confirms forested wetlands as interesting, hotspots of yeasts with useful properties for treating dye-colored wastewaters, and highlights the importance of the conservation of these ecosystems, which are particularly fragile and vulnerable to variations in precipitation and temperature.

## Electronic supplementary material

Supplementary Information

## Data Availability

The datasets generated during and/or analyzed during the current study are available from the corresponding author on (reasonable) request.
