# Supplementary material for: Mediterranean forested wetlands are yeast hotspots for bioremediation: a case study using azo dyes
Source: Sci Rep. 2018 Oct 29;8:15943. doi: 10.1038/s41598-018-34325-7 (PMC6206003; doi:10.1038/s41598-018-34325-7)
Supplement: Supplementary file 1 — Supplementary Information [file 41598_2018_34325_MOESM1_ESM.docx]

**Electronic Supplementary Information (ESI) for**

**Mediterranean forested wetlands are yeast hotspots for bioremediation: a case study using azo dyes**

Ana C. Sampaio^1^, Rui M.F. Bezerra^1^ and Albino A. Dias^1^

^1^Departamento de Biologia e Ambiente (DeBA), Centro de Investigação e de Tecnologias Agro-ambientais e Biológicas (CITAB), Universidade de Trás-os-Montes e Alto Douro (UTAD), Quinta dos Prados, 5001-801 Vila Real, Portugal. E-mail: asampaio@utad.pt

**Table of Contents**

Table S1 - Physicochemical parameters of superficial water in the forest wetland where the yeasts isolates were collected.......................................................................................2

Table S2. Dye chemical structures and color indexes (C.I.). ..............................................3

| **Parameter** | **January - May** |
| --- | --- |
| pH | 6.3-6.5 |
| Temperature (ºC) | 10.9-15.6 |
| Dissolved O_2_ (mg/L) | 3.7-5.3 |
| Conductivity (µS/cm) | 165.0-231.0 |
| Redox Potential (mV) | 136.0-173.0 |
| NO_3_^-^ (mg/L) | 5.3-8.2 |
| NH_4_^+^ (mg/L) | <0.05 |
| NO_2_^-^ (mg/L) | 0.003 |
| PO_4_^3-^ (mg/L) | 0.10 |

Table S1 - Physicochemical parameters of superficial water in the forest wetland where the yeasts isolates were collected

| **Dye** | **C.I.** | **Chemical structure** |
| --- | --- | --- |
| Reactive Black 5 (RB5) | 20505   | 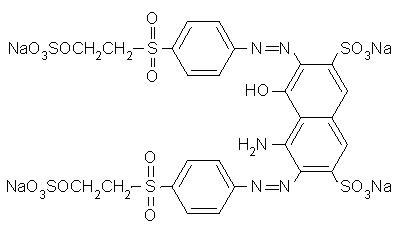 |
| Reactive Orange 16 (RO16) | 17757   | 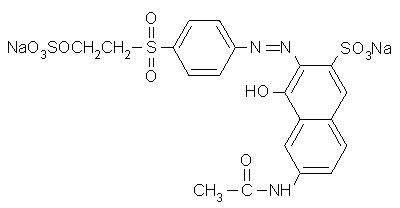 |
| Reactive Violet 5 (RV5) | 18097   | 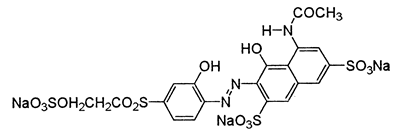 |
| Methyl Orange (MO) (also known as Acid Orange 52) | 13025   | 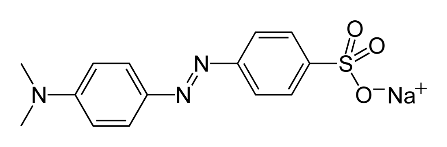 |
| Acid Red 57 (AR57) | 18097   | 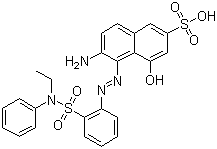 |

Table S2. Dye chemical structures and color indexes (C.I.).
